# Snoezelen: Children with Intellectual Disability and Working with the Whole Family

**DOI:** 10.1100/tsw.2004.105

**Published:** 2004-07-03

**Authors:** Karim Nasser, Carmit Cahana, Isack Kandel, Shlomo Kessel, Joav Merrick

**Affiliations:** ^1^ Lev Hakadosh, Residential Care Center for Children, Haifa, Israel; ^2^ National Institute of Child Health and Human Development, Office of the Medical Director, Division for Mental Retardation, Ministry of Social Affairs, Jerusalem, Division of Pediatrics, Ben Gurion University, Beer-Sheva, Israel; ^3^ Faculty of Social Science, Department of Behavioral Sciences, Academic College of Judea and Samaria, Ariel, Israel; ^4^ Sarah Herzog Children Village, Afula, Israel

**Keywords:** mental retardation, developmental disability, intellectual disability, human development, public health, Snoezelen, Israel

## Abstract

Snoezelen, or controlled multisensory stimulation, was first introduced in Israel in 1993. This paper presents a new concept of working with the whole family in the Snoezelen room with the participation of a social worker. The purpose was to facilitate family encounters with the child, to enable parents and siblings to become better acquainted with the resident through his/her strengths and special abilities, to encourage parental involvement in the care, to encourage increased visits, to improve quality of life (QOL) for the resident, and to reinforce a better relationship between resident, family, and home. Sessions were divided into two major parts. The first segment (duration 20—40 min) was free activity and the second was more structured (duration 15—30 min). Case stories are presented to illustrate the positive effects of this approach. Snoezelen can be used with the entire family with the participation of a social worker and can add new dimensions to communication.

### INTRODUCTION

The concept of “Snoezelen”, or controlled multisensory stimulation, was introduced by staff working at two Dutch centers for persons with intellectual disability (ID) in the 1970s[1,2]. The word “Snoezelen” is a combination of two Dutch words: *snuffelen* (the sniffing of a dog) and *doezelen* meaning to slumber, relax, or doze[1,2]. Snoezelen refers to a specially equipped room(s) where the nature, quantity, arrangement, and intensity of stimulation is controlled[2] with an environment designed to stimulate the senses by means of light, sound, touch, smell, and taste[3].

The founders of the concept[1] used the method as a leisure or relaxation activity without therapeutic elements or supervision, and initially resisted any notion of research in the fear that it would become more therapeutic, objective, and product oriented[4]. Over time though, this has changed and a recent review[4] identified 21 research studies, where 14 studies involved persons with ID and 7 studies involved people with dementia. Of these studies, 14 reported positive effects within the Snoezelen room session, 4 showed positive postsession effects, and 2 had long-term effects and one was inconclusive.

Snoezelen was first introduced in Israel within a day-treatment center for children with ID in 1993[2,5] and the first Snoezelen room within the Division for Mental Retardation (DMR) was established at one of the residential care centers in 1995. Today in Israel, this method is used in more than 25 residential care centers and 3 community settings for persons with ID. Since the year 2000, a part-time physiotherapist has been employed to supervise the treatment and development of the method nationally for the DMR. Professional meetings among the staff take place every 4 months and a certification course has been established on a national basis for the different professionals involved (occupational therapists, physiotherapists, teachers, musical therapists, nurses, speech therapists, or caregivers). The first handbook[2] for practitioners was published in 2002.

Also in the year 2000, the Ministry of Education introduced Snoezelen as a treatment concept for students enrolled in special education programs around the country, and recently several nursing homes for the elderly and rehabilitation institutions for the disabled have taken up the method. The major medical center for children in Israel, Schneider Children’s Medical Center, has also established Snoezelen in their surgical department.

The concept of Snoezelen was introduced in residential care centers for persons with ID in Israel in order to add to the variety of treatment possibilities for this population. It was seen as another instrument for communication between therapist/caretaker and client. The concept also made it possible for multidisciplinary interaction between caretakers and professionals.

In one residential care center for children with ID, the occupational therapist started work with the social worker within the Snoezelen room, which opened new horizons for the treatment of the entire family. The purpose of this presentation is to present one novel concept of working with the whole family.

### METHODS

In contrast to Holland, where the primary use of Snoezelen has been relaxation and leisure time[1], the focus in Israel from the start has been on treatment and therefore assessment of the client before, during, and after treatment in the Snoezelen room. The client has the choice of which environment in the room he or she wants, but the therapist is there to activate the client so that the time spent in the room is active time. The time in the Snoezelen room is usually limited to 30 min and most treatments today are performed on a one-to-one basis. The Lev Hakadosh, Residential Care Center for Children in Haifa decided on a family approach.

#### Study Design and Goals

The Snoezelen room at Lev Hakadosh started operation in July 1999 and each resident was evaluated as to his/her function and reaction to the different stimuli in the room. In October 2000, it was decided to involve the parents in the experience and invite them to participate in the sessions. This worked well until the day one whole family arrived (parents and three siblings) and all wanted to participate in the session. The staff adapted quickly and it turned out to be a most spectacular session. On the basis of that experience, we decided on a social work project to work with the family.

The goals were to facilitate a family encounter with the child with ID, to enable parents and siblings to become better acquainted with the resident through his/her strengths and special abilities, to provide family leisure activity, to encourage parental involvement in the care, to encourage more visits, to improve quality of life (QOL) for the resident, and to reinforce a better relationship between resident, family, and home.

Another goal of this study was to review the literature on social work interaction through the Snoezelen method.

### Participants

Lev Hakadosh or Sacre Coeur, Residential Care Center for Children in the city center of Haifa, operating under the authority of the Catholic Church, is funded and under supervision of the DMR of the Ministry of Social Affairs. Within the center, there is a total of 61 residents: 31 (17 boys, 14 girls) aged 0–9 years, 28 (15 boys, 13 girls) aged 10–19 years, and 2 (1 male, 1 female) aged 20–22 years. All residents are patients with either severe or profound levels of ID, who require nursing treatment; 8 have nasogastric tube, 15 percutaneous endoscopic gastrostomy (PEG), 2 have Down syndrome, 42 suffer from epilepsy, 4 are blind, and all are wheelchair bound. In the family social work project, all families were invited to participate.

The staff involved consisted of the occupational therapist and social worker.

### Procedures

Initial contact with the families and schedules for family visits were facilitated through the social worker, who also worked together with the occupational therapist in the Snoezelen room with the entire family.

The sessions were divided into two major parts. The first part (duration 20–40 min) was free activity with a happy, active, and playful atmosphere with visual effects and music. The second part was more structured (duration 15–30 min) with a relaxed, quiet atmosphere with appropriate light and music. In the first part, the children played, laughed, and enjoyed themselves together with their sibling with ID and the role of the occupational therapist was to guide the activities, while the social worker participated, answered questions from the parents, or addressed emotional issues that came about. In the second part, the family lied down, relaxed, and massaged the child with ID with oils and often the siblings also requested massage. The music was more energetic in the first part, while more slow and magical in the second part.

In order to review the literature on Snoezelen and the social work approach, we conducted computerized searches of Medline/PubMed, Social Science Information Gateway (SOSIG), Scirus, APA on-line, PsychCrawler, Ingenta, World Wide Web Resources for Social Workers, and Infotrieve.

## RESULTS

### The Family Project

From December 2000–June 2002 (18 months), we had a total of 210 family sessions (average of 11.7 session per month) with a total of 47 families who participated (77% of the 61 families); 31 families had 1–5 sessions, 12 families participated in 6–9 sessions, and 4 families had 10 or more (range 1–21 visits). In contrast, for the proceeding 12 months before the project started, parents visited on an average of four visits per month.

Since this project worked without a formal research protocol, much of the resulting data was gathered through conversations with the parents, siblings, and discussions among the professionals.

The parents expressed satisfaction with the sessions; they enjoyed seeing their child smiling, active, and happy and having the opportunity to interact with the child and the siblings in a relaxed and somewhat magical atmosphere. The parents also reported that the siblings talked about the room at home, asked to visit more often, and were very excited on the days when they knew they would visit our center. The siblings were always very excited and disappointed when the sessions ended. They said the room was fun and asked when they could come again. The child with the disability could experience more intimacy with the parents and siblings, more body contact and manipulation.

Among the professionals, we realized that the room facilitated a more relaxed interaction with the parents, who often revealed intimate emotional discussions about the earlier experiences — their fear, grief, and sadness about having a child with disability — which opened up new opportunities for therapeutic conversations and support.

Several case examples can illustrate the positive effects of the Snoezelen room in this setting:

- One mother had experienced problems with touching her disabled daughter since birth, but with support and help, she realized that it was possible to receive and give closeness and warmth to her child in this relaxed atmosphere.
- Another family had visited their child infrequently and not at all for more than 6 months. When they were invited and came, they experienced a very close contact with their child, who then died a few days later due to serious health problems. The family expressed gratitude for having had the opportunity to part with their child this way and will always remember this last visit with a positive feeling.
- One family had never dared to have their child home for visits in the 3 years he was in the residential care center due to fear from the difficulties at home prior to placement. After a few months of sessions, where we worked with them on this issue, they took him home for 3 days and described a relaxed and happy visit at home with the siblings and extended family. This enabled the family to now have the child at home on a regular basis.
- A single mother arrived for weekly sessions and experienced her child alert, responsive, and warm in the room with her. These sessions gave her an important memory of her relationship with her child when the child died 6 months later.

#### The Computer Search (May 2004)

The search of Medline/PubMed identified a total of 23 studies. Nine studies involved Snoezelen and older persons or persons with dementia, seven concerned chronic pain management, four concerned persons with ID, one was a general paper, one was of behavior effects, and one was on a pediatric intensive care unit. Social or family work approach was not found.

The Social Science Information Gateway (SOSIG) had no information on Snoezelen, while Scirus had 31 journal results and 363 web results, but none on social or family work.

APA on-line, PsychCrawler, and World Wide Web Resources for Social Workers had no results on Snoezelen at all, while Ingenta found 7 papers and Infotrieve 53 papers, but none on social or family work.

### DISCUSSION

In recent years, there have been a few reviews on research studies and Snoezelen. Schofield and Davis[6] from Sheffield, U.K. reviewed the literature from the nursing perspective concerning sensory deprivation research. The issue of sensory deprivation dates back to the 1950s, but in fact had already been highlighted by the nursing pioneer Florence Nightingale (1820–1910) a century before[6]. This review combined sensory deprivation, or rather sensory restriction, with chronic pain and suggested Snoezelen as a treatment tool[6], which resulted in a study of 73 patients with chronic pain[7]. Their study compared

Snoezelen with a traditional relaxation program and found that patients who experienced the Snoezelen environment did slightly better than the control group in terms of pain and self-efficacy[7].

Snoezelen for persons with dementia has been the focus of research by the School of Nursing at Deakin University, Australia[8,9], where 25 older persons with dementia were studied in the Snoezelen room, in a landscaped garden, and in normal living room activity. Their study involved the use of both qualitative and quantitative methods. The quantitative data indicated that the garden and the Snoezelen room were associated with an increase in the ratings of pleasure, but did not suggest any benefit of these two environments over that of the living room, when one-on-one care was given. The interviews with staff and visitors, on the other hand, revealed pleasure experienced by participants in the garden and Snoezelen room. There was no evidence that one environment was better than any other, but that all improved effect. Differences were found between the qualitative and quantitative components of the study with the qualitative data showing that the garden and Snoezelen room both improved effect in different ways and for different reasons. The qualitative data also gave clear indication that the Snoezelen and garden environments were enjoyed by the caretakers and visitors just as much as the residents.

A recent Cochrane review[10] was performed with older persons with dementia. The two studies reviewed[11,12] examined the short-term effect of Snoezelen, but the Cochrane review could not conclude anything about the effects due to limited data, different methodology, and control conditions. The authors of the review therefore appealed for further systematic and scientific studies in order to examine the clinical value of Snoezelen for persons with dementia.

Studies on Snoezelen in persons with ID were recently reviewed[4] and 14 studies had taken place within this population. Studies have been both with children and adults with moderate to severe and profound ID and the scientific evidence of positive effects of this treatment should be taken with some caution. The option of Snoezelen in a population with few or limited treatment solutions has made this a popular approach. The majority of the studies reviewed[4] showed a positive effect within the sessions, but long-term effects have not been proven. Many studies have methodological concerns due to the population studied, lack of controls, limited number of sessions, and the use of qualitative or descriptive data.

Our project was also limited in a number of ways. It was initially a clinical project and not a research project. The project evolved according to events taking place with the families. There are no hard data to support a hypothesis, but only the descriptive results from our own observations, case reports, and interviews with the parents and siblings. The novel part of this study, to our knowledge and the literature search, is the participation of the entire family with siblings in the Snoezelen room during sessions and in addition the new possibility for social workers to participate in the room. This new approach will make the social worker see the client and the family in a complete new and different environment. We found that the communication with the family was facilitated in a positive way, emotional issues not ventilated before by the parents came up and it also opened the possibility for the family to see their child in a more positive way.

From the literature search and our contacts at international meetings, we find it interesting to note that the professionals in Holland involved with Snoezelen were from the nonmedical professions, in the U.K. and Australia from the nursing profession, the U.S. from psychology, and in Israel from physiotherapy and occupational therapy. Through this project, social workers have been introduced to Snoezelen.

### ACKNOWLEDGMENTS

The authors would like to thank the children and their parents of the Lev HaKadosh for their cooperation.

### BIOSKETCHES

**Karim Nasser, OT**, is working at the Lev Hakadosh, Residential Care Center for Children in Haifa, Israel with interest in the Snoezelen method, occupational therapy, and intellectual disability. E-mail: soad1@netvision.net.il

**Carmit Cahana**, **MScPT**, is the chief physiotherapist at the Office of the Medical Director, Division for Mental Retardation, Ministry of Social Affairs, Jerusalem, Israel. Her research interests include the role of the physiotherapist and persons with intellectual disability, assistive technology, Snoezelen or sensory multistimulation, and service utilization. E-mail: carmit_c@netvision.net.il

**Isack Kandel, MA, PhD,** is Senior Lecturer at the Faculty of Social Sciences, Department of Behavioral Sciences, the Academic College of Judea and Samaria, Ariel. During the period 1985–1993, he served as the Director of the Division for Mental Retardation, Ministry of Social Affairs, Jerusalem, Israel. E-mail: Kandeli@aquanet.co.il

**Shlomo Kessel, MSW**, is currently the Director of the Sarah Herzog Children's Village in Afula, Israel. Formerly, he was the Director of a residential care center for persons with intellectual disability with interests in aging, prevention of falls, and service provision. E-mail: shlomo@emunahafula.org

**Joav Merrick, MD, DMSc,** is Professor of Child Health and Human Development affiliated with the Zusman Child Development Center, Division of Pediatrics and Community Health at the Ben Gurion University, Beer-Sheva, Israel; the Medical Director of the Division for Mental Retardation, Ministry of Social Affairs, Jerusalem; and the Founder and Director of the National Institute of Child Health and Human Development. He has numerous publications in the field of child and human development, rehabilitation, intellectual disability, disability, health, welfare, abuse, advocacy, quality of life, and prevention. Dr. Merrick received the Peter Sabroe Child Award for outstanding work on behalf of Danish Children in 1985 and the International LEGO-Prize ("The Children's Nobel Prize") for an extraordinary contribution towards improvement in child welfare and well being in 1987. E-mail: jmerrick@internet-zahav.net. Website: www.nichd-israel.com
